# Translation, cross-cultural adaptation and preliminary validation of the Dutch version of the TOGETHER questionnaire - a tool designed to assess how family members of patients perceive their involvement in the care provided by nurses

**DOI:** 10.1016/j.ijnsa.2026.100635

**Published:** 2026-07-20

**Authors:** Thom J.T. Westdorp, Jolanda M. Maaskant, Julie Cussen, Michael. J. Ireland, Andrea P. Marshall, Anne M. Eskes

**Affiliations:** a Amsterdam UMC location University of Amsterdam, Department of Surgery, Meibergdreef 9, Amsterdam, the Netherlands; b Amsterdam UMC location University of Amsterdam, Epidemiology and Data Science, Meibergdreef 9, Amsterdam, the Netherlands; c Amsterdam UMC location University of Amsterdam, Emma Children’s Hospital, Meibergdreef 9, Amsterdam, the Netherlands; d Amsterdam UMC location University of Amsterdam, Department of Internal Medicine, Meibergdreef 9, Amsterdam, the Netherlands; e School of Nursing and Midwifery, Griffith University, Gold Coast, G01 2.03 Griffith University, QLD 4222, Australia; f Faculty of Health, Center of Expertise Urban Vitality, Amsterdam University of Applied Sciences, Amsterdam, the Netherlands; g Gold Coast Hospital and Health Service, Southport, Queensland, Australia; h School of Psychology and Wellbeing, the University of Southern Queensland, Springfield, Queensland, Australia

**Keywords:** Family-centered nursing, patient care, nursing care, Nursing Staff, Hospital, hospital departments, primary health care, factor analysis

## Abstract

**Background:**

Family-centered care improves knowledge, satisfaction and well-being for patients and families. However, it is still insufficiently implemented in adult hospital care even though family want more involvement. Understanding their experiences is essential to improve the collaboration between family and nurses.

**Aim:**

To explore the translation, cross-cultural adaptation, structural validity, and reliability of the TOGETHER questionnaire within the Dutch context. The TOGETHER questionnaire is a tool designed to assess how family members of patients perceive their involvement in the care provided by nurses.

**Design:**

Cross-sectional data collection of the TOGETHER questionnaire.

**Setting:**

19 nursing wards of a Dutch university hospital

**Participants:**

A total of 286 family members of patients that were admitted to a participating hospital ward were included

**Method:**

The 23-item questionnaire was translated and assessed by five family members and two healthcare professionals prior to distribution. Family members of adult patients admitted to a university hospital ward were asked to complete the questionnaire. Structural validity was assessed using exploratory factor analysis, and reliability was evaluated with Cronbach’s alpha.

**Results:**

In total 286 family members completed the questionnaire. Six items were removed because of low correlations or cross-loadings on multiple factors. The resulting structure of the questionnaire consisted of 17 items, loading onto one of three factors (Collaboration, Involvement and Communication), together explaining over half of the variance. All the factors demonstrated good internal consistency.

**Conclusion:**

The exploration of the Dutch adaptation of the TOGETHER questionnaire suggests strong psychometric properties. The questionnaire can be used to evaluate the experiences and perceptions of family members regarding their involvement in care by nurses, enabling hospital wards to identify strengths and areas for improvement in the delivery of family-centered care**.**

**Registration:**

not registered.

**Patient or public contribution:**

Family members of patients admitted to the nursing ward and healthcare professionals evaluated the clarity and relevance of the translated questionnaire.

**What is already known**

• The involvement of family in care has positive effects on family members and patients.
• The involvement of family in care is far from implemented in adult care.

Alt-text: Unlabelled box dummy alt text

**What this paper adds**

• This study explored the structure of a questionnaire that provides nursing wards with insight into how they involve family members in care.
• Using this questionnaire can support nursing wards in promoting and facilitating family centered care.

Alt-text: Unlabelled box dummy alt text

## 1 Introduction

Family-centered care is a model of care with a long history. It has been an integral part of pediatric care since the 1950s (Shields, 2010), and subsequently expanded to adult care (Gerteis et al., 1993). In recent years, there has been an increased expectation for involvement of family members in the care of hospitalized adult patients (Kwame & Petrucka, 2021). Family-centered care provides family members with greater confidence, increases their knowledge, enhances their satisfaction with the care provided, and improves their caregiving skills (Park et al., 2018). In addition, it has a positive effect on family support, readiness for discharge, shared decision-making, and reduces the likelihood of anxiety and depression (Garrouste-Orgeas et al., 2016; Huang et al., 2022; Vardanjani et al., 2021). The presence of family members during invasive procedures or resuscitation can reduce stress and anxiety (Vardanjani et al., 2021), while active involvement further benefits patients by reducing anxiety and improving self-care skills, knowledge, health management, and quality of life (Leino-Kilpi et al., 2016; Van De Graaff et al., 2018).

## 2 Background

However, despite the evidence that family-centered care in hospitalized adults enhances patient outcomes, it is still not fully implemented across all aspects of hospital care (Thirsk et al., 2021). Family members feel the need to be involved to bridge the knowledge between the patient and the healthcare professionals (Hemle Jerntorp et al., 2025). To facilitate effective collaboration with the interprofessional team, family members need caregivers who are open, friendly, patient, approachable and honest, and who are proactive in seeking and maintaining contact with them (Wittenberg et al., 2018). Many family members report that they are less involved in the care of their loved ones than they would prefer and that they experience difficulties contacting nurses when they need information or assistance (Bello et al., 2023). Additionally, family members report insufficient support from healthcare professionals during shared decision-making and in preparing for the post-hospital phase (Woldring et al., 2025). There is an urgent need to better understand when and where family members feel excluded from their relative’s care, so that nurses can better facilitate and improve family-centered care (Leino-Kilpi et al., 2016).

Although Dutch nurses generally hold positive attitudes toward involving family members in care (Bloemberg et al., 2026; Maaskant et al., 2022), hospital strategies and policies need to be further developed to formally recognize family members as important partners of the healthcare team (Woldring et al., 2025). The Dutch Association of Hospitals, a national organization representing hospitals and hospital care providers in the Netherlands, published a report stating that the involvement of family members is not yet standard care and that experiences are not systematically measured (Nederlandse Vereniging van Ziekenhuizen., 2026).

Many hospitals use standardized patient monitors through which patients can express their healthcare experiences (Al-Abri & Al-Balushi, 2014). However, the experiences of the patient’s family members with the healthcare are often overlooked (Kleefstra et al., 2015). Given the need for a fundamental shift in hospital care, including a more active role of families (Eskes et al., 2023), it is essential to evaluate how families experience the care delivered. The initial development of an instrument to evaluate experiences of family members with their involvement in nursing care of hospitalized patients was executed in a collaboration of an Australian and Dutch research team. Since the Netherlands has, on average, the shortest hospital stays in Europe and family members often assume the role of primary caregivers at home (Cai et al., 2021; Eurostat, 2023), it is crucial to be able to assess the experiences of family members with hospital care, specifically in the Dutch context. Therefore, our aim was to establish the translation, validation, and the reliability of this questionnaire in the Dutch context to ensure its cross-cultural adaptation.

## 3 Method

### 3.1 Design and participants

A cross-sectional study was conducted in a large university hospital in the Netherlands (Amsterdam UMC, Amsterdam) between February and April 2024. Amsterdam UMC has 819 beds located on 39 clinical wards that provide highly specialized clinical care. Each ward has approximately 15 to 25 beds with a nurse-to-patient ratio of either 1:4 or 1:5. Visiting hours range from 5 to 9 hours daily across wards, which is when most contact between healthcare providers and family members occurs. Of the 39 wards, 19 participated in the study. Family members of patients admitted to one of the participating wards were eligible for participation in the study. We used a broad definition of family, i.e., a self-defined group of individuals, considered important to the patient, regardless of blood or legal ties, such as siblings, friends, and neighbors.

The following inclusion criteria were used: (1) family member aged ≥18 years; (2) sufficient Dutch language proficiency to fill out the questionnaire; (3) ≥2 visits during admission; and (4) provision of informed consent. Family members of patients admitted to the intensive care unit, medium care unit or cardiac care unit were excluded.

### 3.2 Instrument

A newly developed tool, the TOwards Gathering Evidence to evaluaTe in-Hospital family partnERships (TOGETHER) questionnaire, was used to investigate family members’ experiences of involvement in nursing care for hospitalized patients. The initial development of questionnaire items was informed by the work of Parmar et al. (2021) and Hengeveld et al. (2021) both of whom identified competencies for family-centered care in the hospital setting. Parmar et al. (2021) developed a collaboratively designed framework that consists of six domains: acknowledging the caregiver’s role; ensuring effective communication; partnering with caregivers; promoting caregiver resilience; supporting navigation of health and social care systems and available resources; and reinforcing a care culture and context that enables these aspects. Hengeveld et al. (2021) identified four key themes of hospital-based family-centered care: nurse-patient-family partnerships; family-centered care as a core component of nursing; prerequisites for family-centered care; and positive attitudes toward family involvement. Together, these authors identified a total of 93 indicators that were considered important to family members regarding family-centered care. After removing duplicates, 51 indicators were converted into statements, which were evaluated for relevance, comprehensibility, and conceptual clarity by the Australian and Dutch research teams, which included healthcare consumers. This rigorous process resulted in the TOGETHER questionnaire as used in this study (supplementary 2). A more detailed version of this process is described elsewhere (Cussen et al., 2026).

The initial version of the TOGETHER questionnaire under investigation consisted of 23 neutral worded statements. The family members could respond to each statement using a 5-point Likert scale: "totally disagree", "disagree", "neutral", "agree" or "totally agree". For two statements related to beliefs, culture, and spirituality, a “not applicable” option was included, as these may not be relevant to every individual. The “not applicable” option was handled as a missing option.

The questionnaire was conceptualized as a reflective measurement model, in which the latent construct of ‘family members’ experiences of involvement in nursing care’ represents an underlying attitude that is reflected in the observed indicators. Changes in the latent construct are therefore expected to result in changes in the indicators, rather than changes in the indicators causing changes in the latent construct. Consequently, the indicators are expected to be correlated, as they represent manifestations of the same underlying construct (Coltman et al., 2008).

In addition to completing the statements of the TOGETHER questionnaire, the participants were also asked to provide demographic information, such as gender, age, relationship to the patient, whether they provided informal care for the patient prior to hospitalization, and their religious or cultural beliefs. The Dutch and Australian factor structures were explored in parallel. Exploratory analyses of the Australian version indicated a bifactor structure consisting of 20 items, with factor 1 representing communication and factor 2 representing collaboration (Cussen et al., 2026).

### 3.3 Translation

The TOGETHER questionnaire was translated by an ISO-certified translation agency. First, an independent native Dutch-speaking person translated the original English questionnaire into Dutch. Next, the questionnaire was back translated by two native English speakers, who are bilingual in English and Dutch, to identify potential inconsistencies (Tsang et al., 2017). The research team then reviewed the translated version for conceptual equivalence; no substantial discrepancies were identified.

### 3.4 Ethical considerations

The Medical Ethical Committee of the Dutch university hospital reviewed the protocol and confirmed the study was exempt from formal ethics review under the Medical Research Involving Human Subjects Act, as it posed no additional burden beyond standard care (No 2023.0717, Date 14-09-2023). Written informed consent was obtained from all participating family members after reading an information sheet detailing the time commitment, the voluntary nature of participation, the right to withdraw, the intended use of the data, and the absence of direct benefits or financial compensation. Informed consent obtained before completing the questionnaire and included permission to share anonymized data in the Datavers repository, where data are available to other researchers upon request. Informed consent was obtained only from the family members and not from the patients themselves.

### 3.5 Cultural adaptation

To conduct the cultural adaptation from the perspective of Dutch family members, we invited five family members of hospitalized patients to participate. In addition, we collected the perspective of two healthcare professionals: one with extensive experience as a researcher and lecturer in qualitative research, and one clinical research nurse with a master’s degree in public health. Both professionals were native English speakers and fluid in Dutch. First, we asked the participants to respond to each questionnaire item. Secondly, using a yes/no response format, they indicated whether each item was clearly formulated and considered relevant. The participants could also provide additional written feedback on each item. Based on the feedback, the research team refined and finalized the questionnaire.

### 3.6 Data collection

A paper version of the questionnaire was distributed to family members of patients admitted at one of the 19 clinical wards. We chose a paper-based questionnaire to ensure inclusion of family members with lower e-health literacy and to achieve a high response rate (Daikeler et al., 2019). Nurses working on the participating wards were responsible for inviting the family members to participate in the study. On each ward, a dedicated nurse supervised the data collection. Before the start of the study, the nurses were informed about the aim of the study, the procedures for obtaining informed consent, and the logistics of the data collection. Completed questionnaires were enclosed in envelopes by the family members and collected in sealed boxes on the wards. The coordinating researcher visited the ward once a week to discuss any problems. Every two weeks an update on data collection progress was provided to the participating wards. Completed questionnaires were manually entered in the Castor Database by the coordinating researcher. A minimum sample size of 230 participants was required to meet the commonly cited criterion of at least 10 participants per item (Anthoine et al., 2014).

### 3.7 Statistical analysis

Analyses were conducted using R (version 4.3.2) with the psych package for exploratory factor analysis and reliability, the package GPArotation for factor rotation and the package lavaan for assessing the validity. Continuous variables are presented as mean and standard deviation (SD) or median and interquartile range (IQR) depending on the distribution of the data. Nominal or ordinal data are presented as frequencies and percentages (%). The “not applicable” option in question 21 and 22 was interpreted as a missing. To assess the missing pattern, LittleMCAR test was performed (p > 0.05 data missing completely at random is plausible). In subsequent analyses, missing data were handled using pairwise deletion. Pairwise deletion was selected because, in the context of exploratory factor analysis, it has been shown to yield results comparable to those obtained using imputation-based approaches (Goretzko, 2022).

To determine the factorability of the data the correlation was assessed. Pearson correlation coefficients between items were calculated and visualized in a correlation matrix to identify the degree of association between pairs of variables, identifying strong correlations (r > 0.8) that might indicate redundancy, as well as low correlations (r < 0.3) suggesting weak association with other items, indicating potential misfit. Sample adequacy was assessed using the Kaiser-Meyer-Olkin measure (KMO), values > 0.7 indicate suitability for factor analysis. Additionally, Bartlett's test of sphericity was assessed whether correlations were adequate for factor analysis, p < 0.05 indicating suitability.

After evaluating the factorability, we conducted an exploratory factor analysis to assess the structural preliminary validity of the Dutch version of the TOGETHER questionnaire. Exploratory factor analysis was chosen because, at the time of analysis, there were no prior assumptions regarding the underlying structure of the questionnaire. Additionally, the questionnaire had only recently been translated into Dutch, further justifying the use of an exploratory approach (Kyriazos, 2023).

Principal axis factoring was used to identify latent factors, with the number of factors determined by Horn’s parallel analysis. Factors were retained when their eigenvalues exceeded the corresponding random eigenvalues. For the random eigenvalue, we used 100 iterations and the 95th percentile as the cut-off. We used oblique rotation (promax), as we expected factors to be correlated, given that they represent dimensions of the same overarching construct (family-centered care). Each item was assigned to the factor with the highest loading score, and items with a weak factor loading (< 0.32) were removed (Tabachnick & Fidell, 2013).

Acceptability of the factor solution was defined a priori by interpretative simple structure: primary loadings ≥ 0.40 with secondary loadings ≤ 0.32 and a difference ≥ 0.10; at least three items with salient loadings per factor; item communalities ≥ 0.30; factor intercorrelations < 0.80 to avoid redundancy; and total variance explained ≥ 50 per cent for a two-factor solution (Pett et al., 2003). Cross-loadings were examined if the difference in loading on the highest and the second highest was < 0.10 to determine whether the item had a meaningful attribution to its factor (Acar Güvendir & Özer Özkan, 2022). Convergent validity of the factor structure was evaluated using standardised factor loadings, with standardised loadings of at least 0.50 indicating adequate convergence of items on their intended construct (Cheung et al., 2024). Discriminant validity was examined by inspecting the latent correlation between two factors and 95% confidence interval; values substantially below 1.00 and confidence intervals that exclude 1.00 were taken as evidence that the factors are empirically distinct.

Following rotation and item assignment, Cronbach's alpha was calculated for each factor to assess internal consistency. A Cronbach's alpha between 0.70 and 0.95 was considered acceptable (Terwee et al., 2007). When an item showed a low corrected item–total correlation (r.cor), the r.drop value was examined to determine whether removing the item would improve the internal consistency of the scale.

## 4 Results

### 4.1 Cultural adaptation

Following the translation, the cultural adaptation of the TOGETHER questionnaire was evaluated by five family members and two professionals. Participants indicated that 17 of the 23 items were clearly formulated. Based on their feedback, six items were revised to improve their clarity and cultural appropriateness.

A total of 286 family members completed the questionnaire. The departments were unable to provide the exact number of family members invited, which precluded the calculation of a response rate. Most participants were partners (51.7%) or children (23.8%) of the patients. Of those included, 156 (54%) were female. The median age of the sample was 57 years (IQR: 45-68). Not many family members had been involved in caring for the patient prior to this hospital admission (72.0%). The demographic characteristics of the participants are summarized in Table 1.

**Table 1:** Demographic data.

	N=286
***Gender: n (%)***	
Male	112 (39.2%)
Female	164 (57.3%)
Prefer not to say	1
missing	9 (3.1%)
***Relation to patient: n (%)***	
Partner/spouse	148 (51.7%)
Parents	28 (9.8%)
Child	68 (23.8%)
Sibling	13 (4.5%)
Friend	7 (2.4%)
Other	14 (4.9%)
missing	8 (2.8%)
***Age: median (IQR)***	57 (45-68***)***
***Current employment: n (%)***	
Full-time	102 (35.7%)
Part-time	58 (20.3%)
Retired	78 (27.3%)
No paid employment	10 (3.5%)
Student	3 (1%)
Prefer not to say	4 (1.4%)
Other	14 (4.9%)
missing	17 (5.9%)
***Self-rated health status: n (%)***	
Excellent	49 (17.1%)
Good	122 (42.7%)
Normal	66 (23.1%)
Moderate	18 (6.3%)
Bad	3 (1.0%)
Prefer not to say	7 (2.4%)
missing	21 (7.3%)
***Prior experience as caregiver for the patient: n (%)***	
No	206 (72.0%)
Yes	62 (21.7%)
Prefer not to say	1
missing	17 (5.9%)
***Connected to a culture***^a^***: n (%)***	
No	210 (73.4%)
Yes	50 (17.5%)
Prefer not to say	14 (4.9%)
missing	12 (4.2%)
***Satisfaction with nursing care: median (IQR)***	9 (8-9)

a supplementary 3 shows the cultural backgrounds that were mentioned by the participants, including the number of participants that mentioned the specific cultural background

### 4.2 Construct validity

The amount of missing data per variable ranged from 0.0% to 63% with item 21 (cultural needs) and item 22 (spiritual needs) having the highest numbers after including “not applicable” as a missing value. A total of 187 participants had one or more values missing, Missing pattern was completely at random (littleMCAR, p = 0.20).

**Fig. 1:**
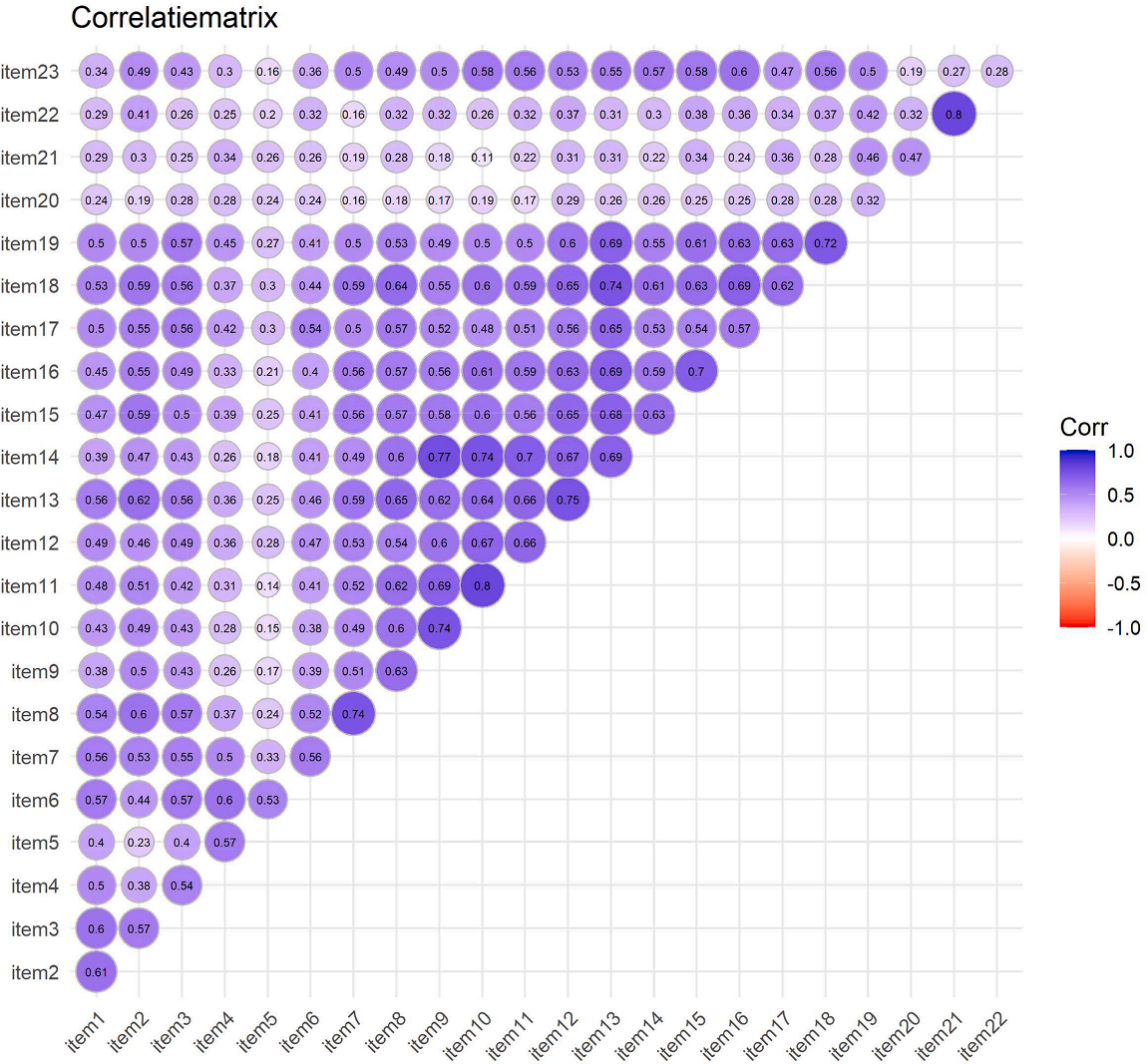
Pearson’s inter-item correlation.

After assessing the correlation (Fig. 1), items 5 (nurses talk in easy to understand way), 20 (visiting according to wishes), 21 (cultural needs), and 22 (spiritual needs) were excluded due to low inter-item correlations (r < 0.30) on almost all other items. KMO indicated excellent sampling adequacy (overall 0.95, all items > 0.95), and Bartlett’s test was statistically significant (χ²(df) = 3562, p < 0.001) supporting factorability.

**Fig. 2:**
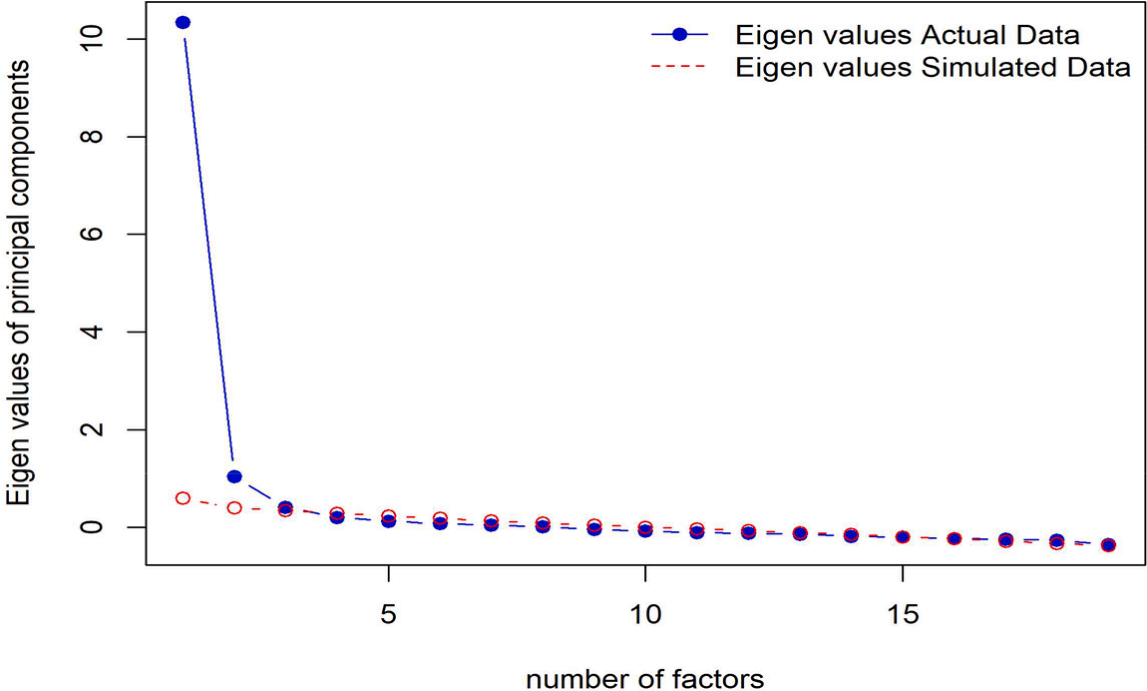
Horn parallel analysis comparing the eigenvalues of the real data (blue) with those of simulated data (red). this initiates that there is a three-factor structure.

Exploratory factor analysis was conducted using the remaining 19 items. Parallel analysis yielded eigenvalues of [10,34; 1,04; 0,41; 0,20] compared to simulated values of [0.58; 0,41; 0,34; 0,28], supporting retention of three factors (Fig. 2). The oblique rotation showed that all items had loading greater 0.30 on the three-factor structure. Two cross-loaders were identified, exhibiting only minor differences in loading between the two factors. Consequently, item 8 (informing without asking) [factor 2 = 0.39, factor 3 = 0.41] and item 12 (comfortable feeling in making decisions) [factor 1 = 0.37, factor 3 = 0.44] were analyzed for their attribution to the questionnaire and subsequently excluded from further analyses. Items removed during analysis are shown in Table 2 and factor loadings are presented in Table 3.

**Table 2:** Items removed due to low correlation (< 0.3) or cross loading.

		Cross-loaders^b^		
Question	Low^a^ correlation	***Factor 1***	***Factor 2***	***Factor 3***
5. The nurses talk to me in a way that is **easy to understand**	**x**			
8. Nurses inform me without me having to ask.			0.41	0.39
12. The nurses make sure that I feel comfortable with being involved in decisions about patient care.		0.44	0.37	
20. The nurses allow me to visit according to my wishes	**x**			
21. The nurses respect my **cultural needs**	**x**			
22. The nurses respect my **religious** and/ or **spiritual** needs	**x**			

^a^ low item–total correlation on many items (< 0.3)

^b^ difference loading between highest and second highest < 0.10

**Table 3:** Results of the Exploratory factor analysis.

	Collaboration (Factor 1)	Involvement (Factor 2)	Communication (Factor 3)	
**Eigen value**	10.34	1.04	0.41	
**Percentage variance explained**	28 %	21 %	16 %	
**Factor loading Questions**				**communalities**
1. The nurses make me feel that what I say is important.	0.23	0.03	**0.57**	0.57
2. The nurses encourage me to share information that I think is relevant with the healthcare team if I want to.	**0.46**	0.09	0.26	0.53
3. The nurses use the information that I provide to improve patient care.	0.37	-0.05	**0.52**	0.60
4. The nurses talk to me in a respectful way.	0.06	-0.08	**0.73**	0.53
6. I feel nurses answer my questions in an honest and accurate way.	-0.12	0.16	**0.81**	0.66
7. The nurses give me information to help me understand what is happening.	0.26	0.18	**0.43**	0.56
9. The nurses ask if I am satisfied with the care provided.	0.02	**0.80**	0.03	0.70
10. The nurses ask me how I would like to be involved in caring for the patient.	-0.01	**0.90**	0.00	0.80
11. The nurses ask me if I would like to be involved in making decisions about patient care.	0.01	**0.80**	0.09	0.72
13. The nurses support me to be involved in caring for the patient.	**0.68**	0.21	0.04	0.75
14. The nurses check that I feel comfortable when helping care for the patient.	0.16	**0.75**	-0.04	0.74
15. The nurses help me to understand the plan of care.	**0.62**	0.20	0.02	0.64
16. The nurses involve the patient and I in planning care and setting goals.	**0.76**	0.14	-0.08	0.68
17. The nurses prioritize goals of care that are important to the patient and me.	**0.52**	0.05	0.26	0.56
18. The nurses make me feel that we are working together to help the patient.	**0.82**	0.04	0.01	0.72
19. I feel the nurses understand the effect the patient’s illness has on me.	**0.85**	-0.11	0.06	0.66
23. The nurses give me information to help me prepare for the patient’s discharge.	**0.42**	0.32	0.00	0.48

The loading of each remaining item on all the factors

The first factor included eight items and was labelled “Collaboration”, referring to items in which nurses help family members understand the care plan (item 15) and support their involvement in patient care (item 13). This factor does not address the initiation of the involvement, but rather the actual fulfillment. The second factor included four items and was labelled “Involvement”, reflecting questions about how family members want to be involved in the care and decision-making and how they feel about being involved (item 10, 11 and 14). It does not cover the tone or accuracy of information exchange, which is represented by factor 3. The third factor includes five items and was labelled “Communication”, as it includes items whether nurses communicate in a respectful way with family members (item 4) and how nurses provide family members information (item 6 and 7). This factor does not pertain to care-specific tasks but instead focuses on the way nurses communicate with family members.

The factors Communication and Collaboration align with the Canadian Medical Education Directives for Specialists competencies Communicator and Collaborator described in Hengeveld et al. (2021) and correspond to the competency domains of communicating with family caregivers and partnering with family caregivers described by Parmar et al. (2021). The factor Involvement corresponds to the competency domain of fostering resilience in family caregivers (Parmar et al., 2021). Factor 1 explains 28% of the variance, Factor 2 explains 21%, and Factor 3 explains 16%. Together, the three factors accounted for 64% of the total variance, which is considered acceptable for psychosocial measurement instrument (Pett et al., 2003).

The convergent validity of the questionnaire can be considered moderate. Six of the eight standardized loadings on Factor 1, all four standardized loadings on Factor 2 and four of the five on factor 3 exceeded 0.50 (Table 3). All item communalities were above 0.50, further supporting the convergent validity of the constructs. The latent correlation of factor 1 and factor 2 was 0.76 (95% CI 0.70-0.80); for factor 1 and 3, 0.63 (95% CI 0.55-0.69) and for factor 2 and factor 3, 0.46, (95% CI 0.36-0.55). As all confidence intervals excluded 1.00, this indicates substantial discriminant validity among the three factors.

### 4.3 Reliability

The reliability analysis indicates excellent internal consistency for Collaboration (α = 0.92) and good internal consistency for both Involvement (α = 0.92) and Communication (α = 0.85). All item-total correlations were good, ranging from 0.69 to 0.86 for Collaboration, 0.70 to 0.77 for Involvement and 0.84 to 0.88 for Communication. We examined whether removing an item would improve the internal consistency of the factors; however, the results indicated this was not the case. The item-total correlations and Cronbach’s alpha values if items were deleted are presented in Table 4.

**Table 4:** Reliability.

	Item correlation	R drop
***Factor 1***	Cronbach’s alpha	(0.92)
Item 2	0.70	0.67
Item 13	0.85	0.82
Item 15	0.80	0.77
Item 16	0.82	0.79
Item 17	0.73	0.70
Item 18	0.84	0.80
Item 19	0.79	0.75
Item 23	0.68	0.65
***Factor 2***	Cronbach’s alpha	(0.92)
Item 9	0.84	0.80
Item 10	0.88	0.85
Item 11	0.84	0.80
Item 14	0.84	0.81
***Factor 3***	Cronbach’s alpha	(0.85)
Item 1	0.74	0.69
Item 3	0.74	0.69
Item 4	0.70	0.64
Item 6	0.77	0.70
Item 7	0.71	0.66

The item correlation reflects the degree to which each item is associated with the total score of its respective factor, while the R drop value indicates the change in reliability (Cronbach’s alpha) if the item were removed.

## 5 Discussion

The aim of this study was to translate, culturally adapt and preliminary validate the TOGETHER questionnaire for use in the Dutch context. The results suggests that the Dutch version of the TOGETHER questionnaire may be a valid and reliable instrument for assessing the family members’ experiences of their involvement in nursing care for hospitalized patients. Exploratory factor analysis revealed a clear three-factor structure (factor 1: Collaboration; factor 2: Involvement and factor 3: Communication), which together explained a substantial proportion of the variance. The original questionnaire was reduced from 23 to 17 items. All three factors demonstrated good internal consistency, suggesting that the questionnaire reliably captures key aspects of family-centered care from the perspective of family members.

The experiences and needs of family members across care settings have mainly been examined in qualitative studies. Many of these studies focus on specific illnesses, specialized care units, or specific situations such as the hospital-to-home transition (Coleman & Roman, 2015; O'Gara et al., 2023). Although qualitative studies are often context-specific, many of the themes they identify are reflected in the items of our questionnaire. For example, Reupert et al. (2023) and Walker et al. (2023) emphasized that information provision, clear communication and caregiver accessibility are important aspects of family experiences. These themes show similarities with items “I feel nurses answer my questions in an honest and accurate way” and “the nurses make me feel that what I say is important” on our questionnaire. In addition, Coleman and Roman (2015) described the hospital-to-home transition, which corresponds to our item “The nurses give me information to help me prepare for the patient’s discharge”.

Several measurements tools have been developed to evaluate family experiences and satisfaction with critical care, such as the Family Reported Experiences Evaluation (FREE) (Wright et al., 2015) and the Family Satisfaction in the Intensive Care Unit 24-item (FS-ICU-24) (Ferrando et al., 2019). Despite differences in target populations, both questionnaires include domains that correspond to our domain Collaboration. The Family-Reported-Experiences Evaluation questionnaire also includes a domain similar to our communication domain. To the best of our knowledge, only one other study has described a measurement tool for family members experiences of patients admitted to non-specialized hospital wards. Drakenberg et al. (2023) developed the Family Involvement in Care Questionnaire based on cognitive interviews. Although the Family Involvement in Care Questionnaire was developed with robust content validity, its clinimetric properties have not yet been evaluated. The Family Involvement in Care Questionnaire comprises 18 items that partially overlap with items of the TOGETHER questionnaire. Both the TOGETHER questionnaire and the Family Involvement in Care Questionnaire assess whether family members are involved in setting care goals, whether they understand the information they receive from the healthcare professional, and whether they are treated with respect. The main distinction is that the TOGETHER questionnaire specifically focuses on nursing care, whereas the Family Involvement in Care Questionnaire addresses the broader healthcare team.

The Dutch version of the TOGETHER questionnaire differs from the Australian version after exploratory factor analysis. In the Australian version, three items 21 (cultural needs), 22 (spiritual needs) and 23 (discharge preparation) were removed due to a high proportion of missing responses. In the Dutch version item 23 was kept, but item 5 was excluded due to low correlation and question 8 and 12 were removed due to cross-loading. The differences in items kept may reflect cultural differences among the questionnaire (Cussen et al., 2026).

We acknowledge several limitations in this study. First, we choose to use a 5-point Likert scale in the development of the questionnaire. A 5-point Likert scale provides respondents with a neutral option, which can be helpful when having difficulty determining their position on an item. However, respondents may also select this neutral option when they do not understand the statement or when they feel that their answer is context-dependent (Nadler et al., 2015). We considered using a 4-point Likert scale with a “not applicable” option as an alternative, because a 4-point Likert scale without a “not applicable” option can increase missing data if participants feel forced to select an answer that does not reflect their experience (Osteras et al., 2008). Thus, the choice of the response scale may have influenced our results. The impact of different response formats on the validity and reliability of the questionnaire remains an important topic for future research.

Second, only a small proportion of our sample identified with a non-Dutch culture (supplementary 3), which limits the cross-cultural validation in the Dutch context. The data in this study were collected in a Dutch city that is highly multicultural with more than 130 different nationalities and ethnicities, and people with diverse ethnic backgrounds make up a significant part of the hospital population (Armengol et al., 2021). As a result, our findings may not fully capture the experiences and needs of ethnic minority families. To ensure broader inclusivity, future research should focus on validating the questionnaire in other languages and among more diverse populations.

Third, we used only paper-based questionnaires, which are known to achieve higher response rates (Daikeler et al., 2019). However, web-based surveys are associated with less missing data and better data quality (Ebert et al., 2018), as well as greater anonymity for sensitive items (Kilinc & Firat, 2017). We attempted to maintain anonymity by minimizing the assistance nurses provided in completing the questionnaire. As the nursing wards distributed the questionnaire to family members, the response rate could not be determined. Therefore, it is important to consider the representativeness of the sample (Fincham 2008; Gustavson et al., 2019). In this study, a heterogeneous sample was obtained, reflecting the diversity of family members of hospitalized patients.

Fourth, the small sample size used during the cultural adaptation of the questionnaire may have influenced the results. It is possible that some items were not interpreted by respondents as intended. Although cognitive interviewing could have been used as an alternative method to assess respondents' understanding of the items, this approach also has limitations. Responses obtained through cognitive interviews may be subject to interpretation by the researcher, which can affect the consistency and objectivity of the findings (Streiner et al., 2015).

Another limitation is the potential for selection and response bias. Nurses may have been reluctant to include family members who were dissatisfied with care. In addition, differences in how individual nurses identified, informed, or encouraged potential participants may have influenced which types of family members were ultimately recruited. Because the TOGETHER questionnaire is a self-report instrument administered during hospitalization, responses may be subject to social desirability or courtesy bias, despite our efforts to ensure anonymity. The location where the questionnaire is completed, whether at home or in a hospital, can also influence the responses (Hameed et al., 2018), especially because nurses were the ones who handed out the questionnaire. There also may have been a self-selection bias, whereby family members who agreed to complete the questionnaire after being approached by nurses may have differed from those who declined participation, potentially being more engaged with healthcare services or holding strong opinions on the topic (Olsen, 2008).

Finally, our findings are limited to non-specialized wards in an academic medical center, and it remains unclear whether the questionnaire is suitable for use in specialized wards, such as the Intensive Care Units, pediatric care, or other healthcare settings.

In this study, we performed an exploratory factor analysis to investigate the structure of the TOGETHER questionnaire. It is essential that future research includes confirmatory factor analysis to validate this structure and ensure its robustness and cultural relevance (Nye, 2022). In addition, further studies should examine the clinimetric properties of the TOGETHER questionnaire in diverse populations and healthcare settings, such as acute care and pediatric wards. Research into additional measurement properties, including measurement error, responsiveness and test-retest reliability, is also required before the validity, reliability, and sensitivity of the TOGETHER questionnaire can be considered fully established. In future research, cut-off values should be determined using a comparable questionnaire or a satisfaction measurement method, such as a numerical rating scale. These cut-off values can then be used to create meaningful categories and enhance the interpretability of the results (Clement et al., 2022). At present, the findings can only be evaluated at the level of individual items, allowing identification of specific areas where improvements are needed.

The TOGETHER questionnaire has the potential to provide valuable insights into the quality of care from the perspective of family members when it is used complementary to the Patient Experience Monitor (PEM) used in Dutch hospitals. Its brevity and ease of administration make it highly suitable for routine use in clinical practice. While many hospitals currently focus on the delivery of care primarily from the patient’s viewpoint, there is a growing recognition of the need to also consider the experiences and needs of family members. Implementing the TOGETHER questionnaire enables hospitals to systematically assess and improve the quality of family-centered care, ensuring that both patients and their families are supported throughout the care continuum.

## 6 Conclusion

This study provides preliminary evidence for the structural validity and internal consistency of the Dutch 17-item TOGETHER questionnaire which comprises three subscales (Collaboration, Involvement and Communication) that measure the experiences of family members of hospitalized patients in the Dutch context. This questionnaire can provide healthcare professionals with a tool for assessing family members' experiences, enabling them to identify areas of strength and opportunities for improvement in delivering family-centered care. Further research should confirm the explored factor structure of the Dutch TOGETHER questionnaire in a divers sample using confirmatory factor analysis. It also is important to investigate the origins of the differences in factor structure in the Australian and Dutch versions of the questionnaire.

## Declaration of generative AI and AI-assisted technologies in the manuscript preparation process

During the preparation of this work the author(s) used ChatGPT in order to exchange views on the R coding and certain wording. After using this tool/service, the author(s) reviewed and edited the content as needed and take(s) full responsibility for the content of the published article.

## CRediT authorship contribution statement

**Thom J.T. Westdorp:** Writing – original draft, Project administration, Investigation, Formal analysis. **Jolanda M. Maaskant:** Writing – review & editing, Supervision, Conceptualization. **Julie Cussen:** Writing – review & editing, Conceptualization. **Michael. J. Ireland:** Writing – review & editing, Methodology. **Andrea P. Marshall:** Writing – review & editing, Conceptualization. **Anne M. Eskes:** Writing – review & editing, Supervision, Conceptualization.

## Declaration of competing interest

The authors declare that they have no known competing financial interests or personal relationships that could have appeared to influence the work reported in this paper.

## Appendix Supplementary materials

Image, application 1

Image, application 2
