# Supplementary material for: Translation, cross-cultural adaptation and preliminary validation of the Dutch version of the TOGETHER questionnaire - a tool designed to assess how family members of patients perceive their involvement in the care provided by nurses
Source: Int J Nurs Stud Adv. 2026 Jul 20;11:100635. doi: 10.1016/j.ijnsa.2026.100635 (PMC13448029; doi:10.1016/j.ijnsa.2026.100635)
Supplement: Supplementary file 1 [file mmc1.docx]

**Supplementary 1: The definitive Dutch translation of the items from the TOGETHER-questionnaire**

| **Samenwerking** | Antwoordopties | | | | |
| --- | --- | --- | --- | --- | --- |
| Vragen | Helemaal oneens | Oneens | Neutraal | Eens | Helemaal eens |
| 1. Verpleegkundigen stimuleren mij om informatie, die ik belangrijk vind, met het zorgteam te delen wanneer ik dat wil. | 1 | 2 | 3 | 4 | 5 |
| 2. De verpleegkundigen **steunen mij** om betrokken te zijn bij de zorg voor de patiënt. | 1 | 2 | 3 | 4 | 5 |
| 3. Verpleegkundigen helpen mij bij het **begrijpen** van de zorg die geleverd wordt (zorgplan). | 1 | 2 | 3 | 4 | 5 |
| 4. Verpleegkundigen **betrekken** de patiënt en mij bij het **plannen** van de zorg en het vaststellen van doelen voor de zorg. | 1 | 2 | 3 | 4 | 5 |
| 5. De verpleegkundigen geven prioriteit aan de zorg (doelen) die belangrijk is voor de patiënt en mij. | 1 | 2 | 3 | 4 | 5 |
| 6. Verpleegkundigen geven mij het gevoel dat we **samenwerken** om de patiënt te helpen. | 1 | 2 | 3 | 4 | 5 |
| 7. Ik heb het gevoel dat verpleegkundigen **begrip hebben** voor de invloed die de ziekte van de patiënt op mij heeft. | 1 | 2 | 3 | 4 | 5 |
| 8. Verpleegkundigen geven mij informatie om me voor te bereiden op het ontslag van de patiënt. | 1 | 2 | 3 | 4 | 5 |
| **Betrekking** | Antwoord opties | | | | |
| Vragen | Helemaal  Oneens | Oneens | Neutraal | Eens | Helemaal Eens |
| 9. Verpleegkundigen vragen of ik tevreden ben met de zorg die wij ontvangen. | 1 | 2 | 3 | 4 | 5 |
| 10. Verpleegkundigen vragen **op welke manier** ik betrokken wil worden in de zorg voor de patiënt. | 1 | 2 | 3 | 4 | 5 |
| 11. Verpleegkundigen vragen mij of ik **betrokken** wil worden bij het nemen van **beslissingen** over de zorg voor de patiënt. | 1 | 2 | 3 | 4 | 5 |
| 12. Verpleegkundigen **vragen** of ik mij op **mijn gemak** voel wanneer ik help met de zorg voor de patiënt. | 1 | 2 | 3 | 4 | 5 |
| **Communicatie** | Antwoordopties | | | | |
| Vragen | Helemaal oneens | Oneens | Neutraal | Eens | Helemaal eens |
| 13. Verpleegkundigen geven mij het gevoel dat wat ik zeg belangrijk is. | 1 | 2 | 3 | 4 | 5 |
| 14. Verpleegkundigen gebruiken de informatie die ik geef om de zorg voor de patiënt te verbeteren. | 1 | 2 | 3 | 4 | 5 |
| 15. Verpleegkundigen praten respectvol met mij. | 1 | 2 | 3 | 4 | 5 |
| 16. Ik heb het gevoel dat verpleegkundigen mijn vragen **eerlijk en nauwkeurig** beantwoorden. | 1 | 2 | 3 | 4 | 5 |
| 17. Verpleegkundigen geven mij informatie, zodat ik op de hoogte ben van wat er gebeurt | 1 | 2 | 3 | 4 | 5 |
