# Supplementary material for: Translation, cross-cultural adaptation and preliminary validation of the Dutch version of the TOGETHER questionnaire - a tool designed to assess how family members of patients perceive their involvement in the care provided by nurses
Source: Int J Nurs Stud Adv. 2026 Jul 20;11:100635. doi: 10.1016/j.ijnsa.2026.100635 (PMC13448029; doi:10.1016/j.ijnsa.2026.100635)
Supplement: Supplementary file 2 [file mmc2.docx]

**Supplementary 2: Original version of the TOGETHER-questionnaire**

| Questions | Answer options | | | | |  |
| --- | --- | --- | --- | --- | --- | --- |
|  | never | rarely | sometimes | often | always |  |
| 1. The nurses make me feel that what I say is important. | 1 | 2 | 3 | 4 | 5 |  |
| 2. The nurses encourage me to share information that I think is relevant with the healthcare team if I want to. | 1 | 2 | 3 | 4 | 5 |  |
| 3. The nurses use the information that I provide to improve patient care. | 1 | 2 | 3 | 4 | 5 |  |
| 4. The nurses talk to me in a respectful way. | 1 | 2 | 3 | 4 | 5 |  |
| 5. The nurses talk to me in a way that is easy to understand. | 1 | 2 | 3 | 4 | 5 |  |
| 6. I feel nurses answer my questions in an honest and accurate way. | 1 | 2 | 3 | 4 | 5 |  |
| 7. The nurses give me information to help me understand what is happening. | 1 | 2 | 3 | 4 | 5 |  |
| 8. Nurses inform me without me having to ask. | 1 | 2 | 3 | 4 | 5 |  |
| 9. The nurses ask if I am satisfied with the care provided. | 1 | 2 | 3 | 4 | 5 |  |
| 10. The nurses ask me how I would like to be involved in caring for the patient. | 1 | 2 | 3 | 4 | 5 |  |
| 11. The nurses ask me if I would like to be involved in making decisions about patient care. | 1 | 2 | 3 | 4 | 5 |  |
| 12. The nurses make sure that I feel comfortable with being involved in decisions about patient care. | 1 | 2 | 3 | 4 | 5 |  |
| 13. The nurses support me to be involved in caring for the patient. | 1 | 2 | 3 | 4 | 5 |  |
| 14. The nurses check that I feel comfortable when helping care for the patient. | 1 | 2 | 3 | 4 | 5 |  |
| 15. The nurses help me to understand the plan of care. | 1 | 2 | 3 | 4 | 5 |  |
| 16. The nurses involve the patient and I in planning care and setting goals. | 1 | 2 | 3 | 4 | 5 |  |
| 17. The nurses prioritize goals of care that are important to the patient and me. | 1 | 2 | 3 | 4 | 5 |  |
| 18. The nurses make me feel that we are working together to help the patient. | 1 | 2 | 3 | 4 | 5 |  |
| 19. I feel the nurses understand the effect the patient’s illness has on me. | 1 | 2 | 3 | 4 | 5 |  |
| 20. The nurses allow me to visit according to my wishes. | 1 | 2 | 3 | 4 | 5 |  |
| 21. The nurses respect my cultural needs. | 1 | 2 | 3 | 4 | 5 | not applicable |
| 22. The nurses respect my religious and/ or spiritual needs. | 1 | 2 | 3 | 4 | 5 | not applicable |
| 23. The nurses give me information to help me prepare for the patient’s discharge. | 1 | 2 | 3 | 4 | 5 |  |
